# Assessing spatial structure in marine populations using network theory: A case study of Atlantic sea scallop (*Placopecten magellanicus*) connectivity

**DOI:** 10.1371/journal.pone.0308787

**Published:** 2024-11-13

**Authors:** Karsten N. Economou, Wendy C. Gentleman, Kira A. Krumhansl, Claudio DiBacco, Daan Reijnders, Zeliang Wang, Devin A. Lyons, Ben Lowen

**Affiliations:** 1 Department of Engineering Mathematics and Internetworking, Dalhousie University, Halifax, Nova Scotia, Canada; 2 Fisheries and Oceans Canada, Bedford Institute of Oceanography, Dartmouth, Nova Scotia, Canada; 3 Institute for Marine and Atmospheric Research Utrecht, Utrecht University, Utrecht, Utrecht, The Netherlands; Central Marine Fisheries Research Institute, INDIA

## Abstract

Knowledge of the geographic distribution and connectivity of marine populations is essential for ecological understanding and informing management. Previous works have assessed spatial structure by quantifying exchange using Lagrangian particle-tracking simulations, but their scope of analysis is limited by their use of predefined subpopulations. To instead delineate subpopulations emerging naturally from marine population connectivity, we interpret this connectivity as a network, enabling the use of powerful analytic tools from the field of network theory. The modelling approach presented here uses particle-tracking to construct a transport network, and then applies the community detection algorithm *Infomap* to identify subpopulations that exhibit high internal connectivity and sparse connectivity with other subpopulations. An established quality metric, the coherence ratio, and a new metric we introduce indicating self-recruitment to subpopulations, dubbed the fortress ratio, are used to interpret community-level exchange. We use the Atlantic sea scallop (*Placopecten magellanicus*) in the northwest Atlantic as a case study. Results suggest that genetic lineages of *P. magellanicus* demonstrate spatial substructure that depends on horizontal transport, vertical motility, and suitable habitat. Our results support connectivity previously characterized on Georges Bank and Mid-Atlantic Bight. The Gulf of St. Lawrence genetic lineage is found to consist of five subpopulations that are classified as being a sink, source, permeable, or impermeable using quality metrics. This approach may be applied to other planktonic dispersers and prove useful to management.

## Introduction

Characterizing the structure of a marine species into spatially-disjunct subpopulations that may exchange propagules or larvae is crucial for understanding marine connectivity and informing management and conservation efforts [1]. Previous approaches use empirical data to assess the genetic spatial structure of a species [2–5] or simulate the transport of spawned larvae through hydrodynamic flow fields to model settlement spatial distributions and reveal connectivity and the relative importance of subpopulations as larval sources and sinks [6]. The simulated dispersal has historically been summarized using connectivity matrices quantifying particle transfer among regions of interest defined *a priori* to assess connectivity, e.g., stocks [7] and Marine Protected Areas [8].

Efforts have been made to characterize spatial structure emerging from patterns in the movement of Lagrangian particles in marine systems, including fluid parcels [9], marine debris [10], and propagules [11]. Using network theory and community detection algorithms such as *Infomap* [12], we may delineate regions that are sparsely connected by particle transfer with one another but demonstrate high internal mixing of particles [11]. In network theory, these regions are called communities [13], and may be understood as fairly independent compartments of a network [14], not to be confused with ecological communities. In the context of larval exchange, community boundaries can be interpreted as semi-permeable barriers to larval transport [11]. With this approach, the synthesis of system characteristics (e.g., hydrodynamic or biological information) produce a synoptic view of emergent spatial structure without requiring regions to be defined *a priori* [15].

*Infomap* has been used to detect communities in networks representing transport processes with different scopes of biological characteristics. For example, [11, 16] applied *Infomap* to networks representing the transport of particles at a fixed depth in the closed-domain Mediterranean Sea and [17] extended this to detect communities in the open-domain Arctic and subarctic oceans. [18] further incorporated species-specific suitable habitat to identify priority sites in support of fisheries management. Still, there is limited research on how community structure detected by *Infomap* changes when the transport network is altered by introducing biological characteristics [19], despite its recent use in studies of marine population dynamics [18, 20–22]. We hypothesize that complex biological characterizations of the particles will strongly impact community structure. In situations where limited information is known of a species, or a species is typecast as having generic traits, the competency of this approach to detect meaningful spatial structure given some extent of modelled biology could be of interest to management [1].

Our objective in this paper is to use the Atlantic sea scallop (*Placopecten magellanicus*) as a model species to study the impact of modelled biology on network community structure. This species is chosen because its rich history of empirical and modelling studies informs our model design and serves as a reference with which to compare results to and ultimately understand the capabilities and limitations of our approach. *P. magellanicus* is a benthic marine bivalve ranging from North Carolina, USA, to Newfoundland, Canada [23]. As one of North America’s most significant commercial fisheries [24], it has been the target of numerous spatial structure assessments. In the northwest Atlantic, three distinct genetic lineages of *P. magellanicus* have been described [25].

*P. magellanicus* adults are primarily sedentary but highly fecund broadcast spawners that produce planktonic larvae with extensive dispersal ability, making transport during the species’ planktonic larval duration the dominant component influencing its spatial structure [5]. Connectivity studies have used a Lagrangian particle tracking framework to simulate the transport trajectories of *P. magellanicus* larvae to help quantify exchange among predefined regions [7, 26–28].

We use particle tracking to simulate the dispersal of particles representing *P. magellanicus larvae*. Particles exhibit vertical sinking and swimming behavior and spawn and settle according to a genetic lineage-specific heterogenous distribution of suitable habitat in the northwest Atlantic. Further, we examine the extent to which spatial structure detected by *Infomap* varies with the inclusion of biological characteristics. We build up to this case of highly descriptive biology by first examining cases where suitable habitat and/or vertical motility are not included in the model to compare effects of hydrodynamics and biological factors on connectivity.

## Methods

### Particle simulation

**Fig 1:**
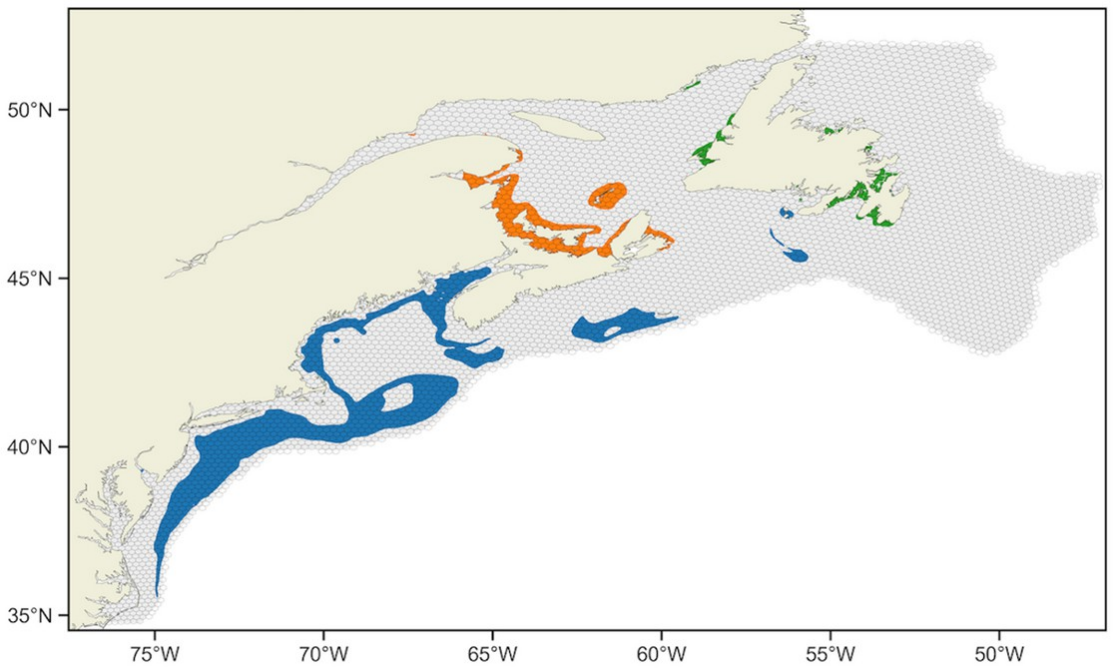
Domain map. Model domain (grey) with the distribution of suitable habitat for the southern lineage (blue), Gulf of St. Lawrence lineage (orange), and Newfoundland lineage (green) [25]. Domain is binned using *H3* resolution index 5.

We use Lagrangian particle tracking to describe planktonic trajectories of *P. magellanicus* as they disperse from spawn to settlement. With this approach, larvae are modelled as point particles in the hydrodynamic flow field, whose transport is calculated based on horizontal advection, horizontal diffusion, and vertical behavior. Our model domain reflects the distribution of *P. magellanicus*, from Cape Hatteras, North Carolina, USA (34.8°N) to Belle Isle, Newfoundland and Labrador, Canada (51.9°N) and bounded in the 510m isobath, chosen to capture the bathymetry of the Cabot Strait in the Gulf of St. Lawrence (Fig 1).

We use climatological monthly outputs averaged over 1990–2015 from the eddy-resolving 1/12° Bedford Institute of Oceanography North Atlantic Model (BNAM) [29] to represent the hydrography of this region. This includes 3D horizontal currents, 3D horizontal diffusivities, mixed-layer depths (MLDs), as well as bathymetries. Horizontal diffusivities were calculated using a modified Smagorinsky scheme [29]. A threshold of 0.01 kgm^−3^ for the density offset relative to the ocean surface is used to calculate local MLDs. Time-varying field data were linearly interpolated assuming model monthly values apply to the middle of each month.

The transport of particles is simulated using the *OceanParcels* Lagrangian framework [30], which performs spatial interpolation of physical fields to particle positions. Our simulations use a 30 min calculation time step with daily output. The horizontal transport of particles is modelled based on advection calculated using the fourth-order Runge–Kutta method and diffusion calculated using the first-order Milstein scheme. We customized *OceanParcels* to run two simulations: (i) buoyant particles maintain a fixed depth of 1 m for the duration of their transport period, in line with previous work [11, 16, 17]; (ii) particles change depth due to simultaneous sinking by gravity and swimming towards a preferred depth centered around the local MLD [31], following the schema outlined by [32]. Particle behavior (i.e., vertical swimming and sinking or maintaining a fixed depth) is assumed to dominate vertical advection and diffusion, so these two physical processes are not included in the simulation.

Individual vertically-motile particles are each assigned a sinking speed and a preferred depth distributed around the local MLD that they swim towards vertically with an assigned maximum vertical swimming speed; they stop swimming if they reach their preferred depth during the time step. The swimming and sinking speeds for each particle are constant throughout dispersal. All distributions follow clipped normal distributions in which random values not within one standard deviation (two standard deviations for depth preference) from the mean are redistributed uniformly to be within this range. Sinking speeds and swimming speeds were estimated to be 0.6(3) mm s^−1^ and 2.4(20) mm s^−1^ respectively for bivalve larvae [33]. Depth preferences and depth initialization were calculated according to a procedure outlined by [32].

Vertically-motile particles that are transported above or below the vertical bounds of the hydrodynamic data during a time step are moved to be just below the modelled surface or above the local bathymetry respectively. Any beached particles (entering a region in which their interpolated velocities are zero, i.e., land) were considered no longer viable, and omitted from post-processing [11, 16].

The model domain (Fig 1) was uniformly seeded with approximately 1.9 million particles (1.3 particles/km^2^) for simulation. An autumn spawn was assumed to occur simultaneously for all particles in mid-September, chosen as it is the most prevalent major spawning period for *P. magellanicus* over the model domain [23, 34, 35]. On September 15, particles are released, and their transport is simulated for the duration of the larvae’s maximum pelagic larval duration (PLD). We approximate the maximum PLD as a constant 45 days. Previous studies [7, 27, 28] have used a slightly smaller PLD for southern regions within our domain for an autumn spawn, but larvae in more northern (colder) regions likely demonstrate a longer PLD than larvae in more southern (warmer) regions [31].

Analysis is done by post-processing the simulations for characterizations of particles that (i) maintain a fixed near-surface (1 m) depth (hereafter called fixed-depth case, abbreviated FD), (ii) seek a preferred depth near the MLD (hereafter called preferred-depth case, abbreviated PD), and (iii) both seek a preferred depth near the MLD and are required to spawn and settle within a heterogenous distribution of suitable habitat (Fig 1) with settlement occurring during a competency period (hereafter called suitable habitat case, abbreviated SH). The same simulation is used for PD and SH so that stochasticity (viz., horizontal diffusion) in particle transport between simulations does not cause discrepancies in results.

The competency period used in the suitable case is assumed as a 7-day period before the maximum PLD of particles during which they can settle [6]. Settlement is assumed to occur on the first day a particle is over suitable habitat during this competency period, a proxy for the ability of larvae to search for appropriate substrate prior to settling [31]. Particles that are never located over suitable habitat during their competency period are considered non-viable larvae and are deleted. In turn, the heterogenous distribution of suitable habitat over the model domain defines viable spawn and settlement locations for the suitable case. Suitable habitat was predicted independently by [25] using species distribution models for each of three genetically-distinct populations, including a southern lineage that spans the Scotian Shelf, Bay of Fundy, Gulf of Maine, and Mid-Atlantic Bight; a Gulf of St. Lawrence lineage; and a Newfoundland lineage (Fig 1).

### Network construction

To construct a network, we first require a grid that tessellates the domain such that any latitude and longitude coordinate of a particle’s position can be assigned to a single grid cell or bin. To achieve this, we use an icosahedral-hexagonal grid composed of hexagons and 12 pentagons over the full globe [17]. We employ the *H3* hexagonal hierarchical geospatial indexing system [36] in a manner described by [37]. H3 supports 16 different resolutions, where we use the resolution 5 index which generates regular hexagons with an average area of 253 km^2^ (average edge length of 8.54 km) and with no pentagons within our domain.

With a discrete description of our spatial domain, each particle’s trajectory can be summarized by a single transition from their spawn (initial or source) bin to their settlement bin to define a flow map [16]. It is imperative to note that this mapping of particle flow retains no information on intermediate bins traversed in particle trajectories between spawn and settlement locations. Therefore, the only particle movement that can be referenced is the abstract transition from spawn bin to settlement bin.

Since the flow map effectively relates directional particle transfer between bins, we can analytically create a network whose topology reflects flow dynamics in the system [38]. Networks are highly flexible mathematical objects composed of discrete objects called nodes that are related by links; they have used extensively to represent and subsequently analyze systems [39, 40]. In a directed and weighted network, each link is oriented with an associated weight to describe the relationship from one node to exactly one node, allowing for asymmetric and biased relationships to be captured by the network. Our transport network is a directed and weighted network whose (i) nodes abstractly represent the set of bins partitioning our domain and whose (ii) directed links represent the directional transitions of particles from one node to another, weighted proportional to the number of transitions [11, 16, 41]. Specifically, the weights of the directed links are normalized to be the probability for a particle spawning at one node to transition to another node [42, 43]. By this token, the network approximates a Markov chain.

Any particles that settle in bins that no surviving particles (i.e., particles that beached or failed to reach suitable habitat to settle in) spawned in are deleted to prevent any nodes without any out-links. This maintains the singly-stochastic nature of the transition matrix, where the sums of outgoing link weights (transition probabilities) from nodes are identically unity and not possibly zero. Such nodes tend to occur around the coast, where a combination of relatively coarse hydrodynamic data, relatively long calculation time steps, and relatively large mixing coefficients results in excessive beaching of particles. Since these nodes are effectively unsuitable habitat (i.e., no surviving particles spawn there), we consistently treat them as unsuitable habitat for settlement.

We produce three transport networks, one for each of the model cases. Each network can be thought of as a particle connectivity matrix describing particle transfer between pieces of domain (bins), i.e., the weighted adjacency matrix (transition matrix) representation of the underlying network [16]. While previous studies have made inferences about connectivity directly from a connectivity matrix of only few regions [7, 27], we use community detection to extract structure inherent to the transport network including hundreds of nodes.

### Community detection

To analyze the flow dynamics occurring in the system, we use the community detection algorithm *Infomap* [12] to partition our network into communities of nodes that are densely intraconnected and sparsely interconnected. *Infomap* is preferable to other algorithms because it utilizes a flow-based approach on the network it operates on, meaning it respects the important weighted and directed nature of the links in our transport networks [12]. Furthermore, *Infomap* detects an unfixed number of communities of varying sizes [16] and is far less restricted by a resolution limit [44] than other techniques [45]. As such, this algorithm has been applied previously to transport networks of marine systems [11, 16–18].

*Infomap* uses a stochastic and recursive heuristic greedy algorithm to partition a network into communities [42]. While *Infomap* can find multi-level solutions (nested community structure) [43], we limit the algorithm to search for two-level solutions (communities of nodes) to maintain a simple interpretation and visualization of structure [46]. We prescribe the scale of spatial structure detected by configuring *Infomap* to set its tuning parameter, called Markov time, equal to two to yield communities that are of an appropriate scale given our bin size, domain size, and PLD [47–49]. We also specify our network as one containing directed links and potentially loops (links that connect a node to itself, caused by a particle settling in its spawn bin). While *Infomap* uses its implemented unrecorded teleportation scheme to move random walkers away from nodes without out-links [50], we choose to completely circumvent including such nodes in our network by manually removing them.

Considering the stochasticity of *Infomap*, we run the algorithm 100 times for each transport network using random seeds 0–99 and proceed with analyses on the seed for each network that best minimizes the map equation [12]: *Infomap*’s internal cost function to evaluate partition quality. We also specify that 20 outer optimization loops should be completed for every random seed Infomap is run for, after which the partition that produces an optimal map equation is recorded [17]. This methodology results in an ensemble of solutions that are similar in quality but potentially degenerate, featuring structurally-distinct topologies [17, 51]. We use a solution that best optimizes the map equation as a representative network partition of the solution ensemble, understanding that there is innate variability in the precise delineations of communities as there is no unique solution to community detection [52]. As a first-order validation of this, the persistence of nodes acting as community boundaries in the solution ensemble is discussed in S2 Appendix, where it is seen that boundaries are drawn quite consistently.

### Quality metrics

Two community quality metrics—the mixing parameter and coherence ratio—have been used to assess the quality of the network partition generated by *Infomap* and ultimately to understand and interpret spatial patterns realized [16, 17]. We employ these metrics but also introduce a third quality metric: the fortress ratio. As discussed below, interpretation of the coherence ratio and fortress ratio together serves as a novel tool for characterizing detected communities in terms of their contribution to connectivity in the system.

The mixing parameter [16] is concerned with evaluating the intracommunity flow of particles. The mixing parameter quantifies how strongly particles are mixed within a community; that is, how uniformly particles flow from each bin in a community to all other bins in that community. The mixing parameter has a magnitude varying between zero and unity, where unity indicates that particles disperse from each bin in a community to every other bin in that community in equal quantities [16].

To evaluate intercommunity flow, the coherence ratio is used [16]. It is mathematically the fraction of particles spawned within a community that settled within that same community and, as such, is a measure of local particle retention. The maximum coherence ratio is unity, occurring when every spawned particle within a community also settled within that same community (i.e., all valid particles were retained). Its minimum value is zero, indicating that all spawned particles in a community settled outside that community. Note that the coherence ratio therefore quantifies intercommunity flow for each community only from the perspective of flow exiting that community.

We define the fortress ratio as the fraction of settled particles within a community that also spawned within that same community. A fortress ratio of unity for a community indicates that every particle that settled within that community was also spawned within that same community. A value of zero indicates every particle that settled in a community spawned outside that community. Note that the complement of the fortress ratio is the fraction of particles settling in a community that spawned in a different community. It immediately follows, then, that the coherence ratio and fortress ratio assess the outward and inward strength of the borders defining a community respectively.

**Fig 2:**
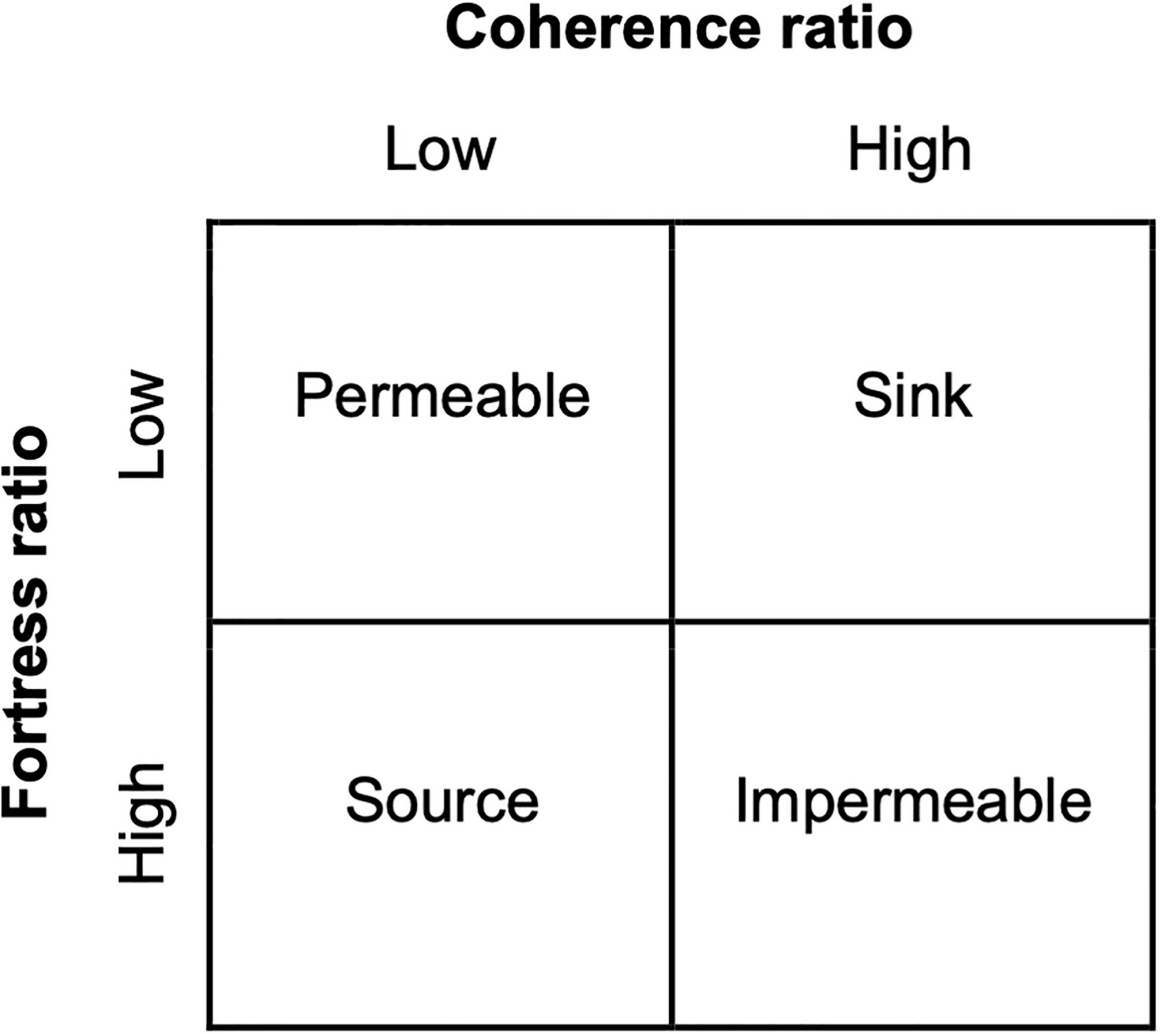
Community classification matrix. Communities are classified based on the magnitude of its coherence ratio and fortress ratio.

When the coherence ratio and fortress ratio are examined in conjunction locally (i.e., for a single community), they can inform the relative particle transfer of that community with all other communities (Fig 2). For example, a community having a high coherence ratio and a low fortress ratio indicates that a large proportion of the surviving particles that spawned in that community also settled there but accounted for only a small proportion of the total number of settled particles in the community. In this case, the community could be classified as a sink community. Conversely, a source community would be classified by a low coherence ratio but a high fortress ratio, indicating a small proportion of retained spawned particles and a small proportion of external particles settling. Thresholds for what magnitudes might be considered “high” and “low” are purposely left undefined to allow for nuance in classifications, e.g., some communities act as larger sinks than others. Communities with strong bidirectional borders (i.e., impermeable or isolated) would have high coherence and fortress ratios.

### Connectivity matrix

For examining specific community-community particle flows, a connectivity matrix can be created as done previously [7, 27], using the communities as regions. Each element on the main diagonal represents the proportion of particle retention for a community, and thus is identical to the coherence ratio of that community. The connectivity matrix, when rows represent spawn community and columns represent settlement community, is necessarily row-stochastic, although the presented precision can be such that the sum of particle transfer proportions for a spawn community is not unity due to rounding.

## Results

### Spatial structure

**Fig 3:**
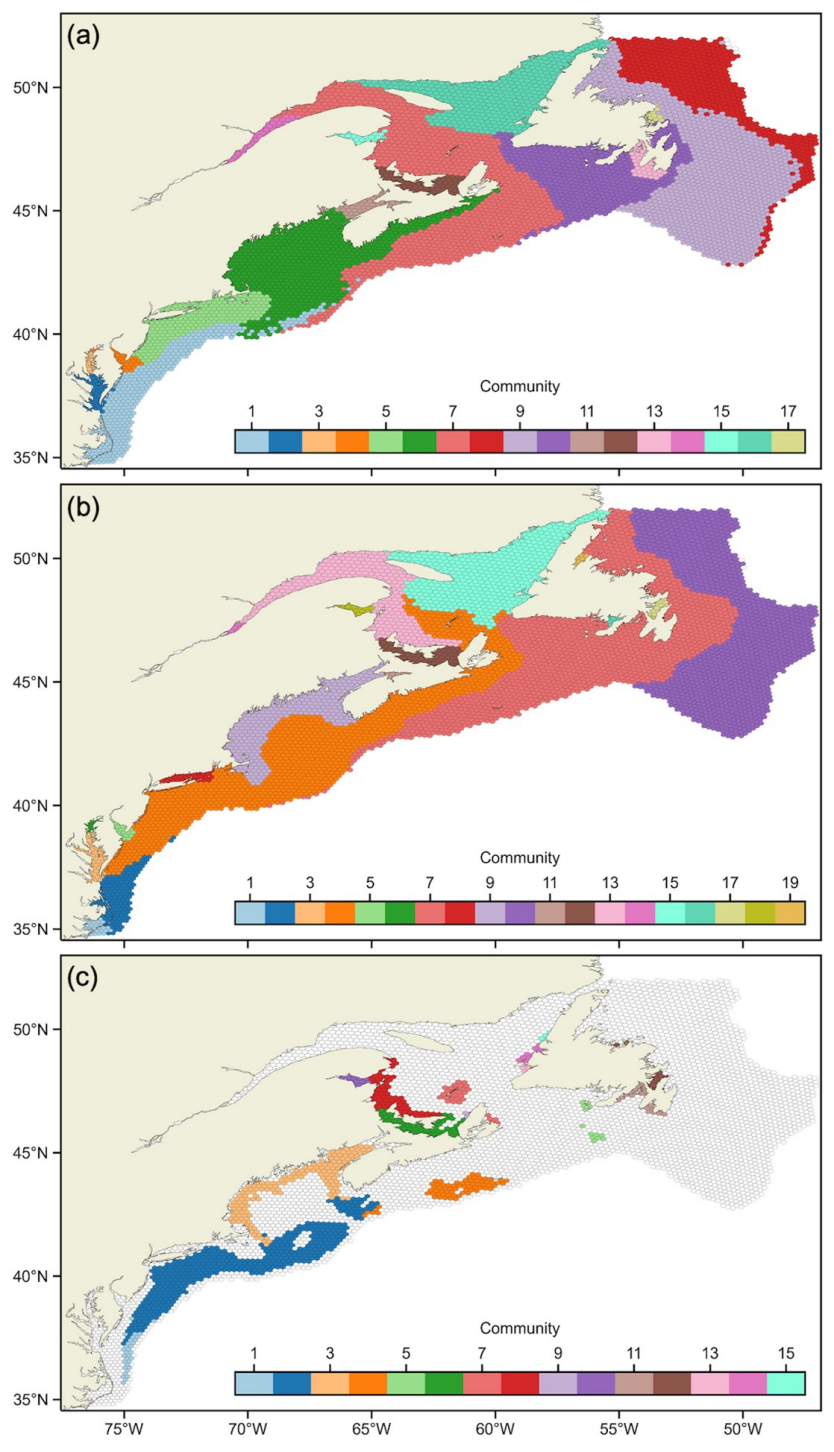
Community structure of transport networks. Cases (a) FD, (b) PD, and (c) SH. Bins are colored by the community to which they belong; those plotted with transparent faces for a particular network were included in the model domain but do not belong to any community as they had no particles that spawned within them survive. Communities are indexed from south to north, within each genetic lineage first if applicable.

Although exhibiting largely different spatial structures (Fig 3), the FD and PD cases contain similar structure at coastal features. This is seen by communities in Northumberland Strait (FD community 12; PD community 12) and in Esquiman Channel and Anticosti Channel (16; 15) having similar delineations. Coastal structure emerging at freshwater-saltwater hydrodynamic interactions in the fixed-depth case (i.e., shallower) extend further out from the head of these coastal features than their PD case (i.e., deeper) analogues. This is seen in Chesapeake Bay (2, 3; 3, 6), Delaware Bay (4; 5), Long Island Sound (5; 8), Bay of Fundy (11; 11), St. Lawrence River (14; 14), and Bay of Chaleur (15; 18).

Some communities in the PD and SH cases have similar delineations such as the Gulf of Maine and Bay of Fundy bounded at Georges Bank and German Bank (PD community 9; SH community 3), Mid-Atlantic Bight (2; 1), Northumberland Strait (12; 6), Bay of Chaleur (18; 10), and southern Gulf of St. Lawrence (13; 8). Also apparent is SH communities that are contained within the boundaries of PD communities. The PD community from Gulf of St. Lawrence wrapping around Scotian Shelf and into Mid-Atlantic Bight (4) entirely contains distinct SH communities in Mid-Atlantic Bight, Georges Bank, and Browns Bank (2); Sydney Bight and Magdalene Islands (7); and Margaree Island (9). The same occurs for the PD community in Esquiman Channel and Anticosti Channel (4) and SH communities in St. George’s Bay (13); Port au Port Bay and Bay of Islands (14); and Bonne Bay (15). Finally, the PD community reaching from Belle Isle to Brown’s Bank (7) contains SH communities in Browns Bank, Western Bank, and Sable Island Bank (4); Saint Pierre and Miquelon and St. Pierre Bank (5); and Placentia Bay and St. Mary’s Bay (11). Interestingly, there is a single instance where a SH community is definitively split between two PD communities, beyond slight differences in delineation: Trinity Bay and Bay of Exploits group together as one SH community (12), but Trinity Bay appears as a distinct PD community (17) and Bay of Exploits groups with a different PD community (7).

### Community quality

**Fig 4:**
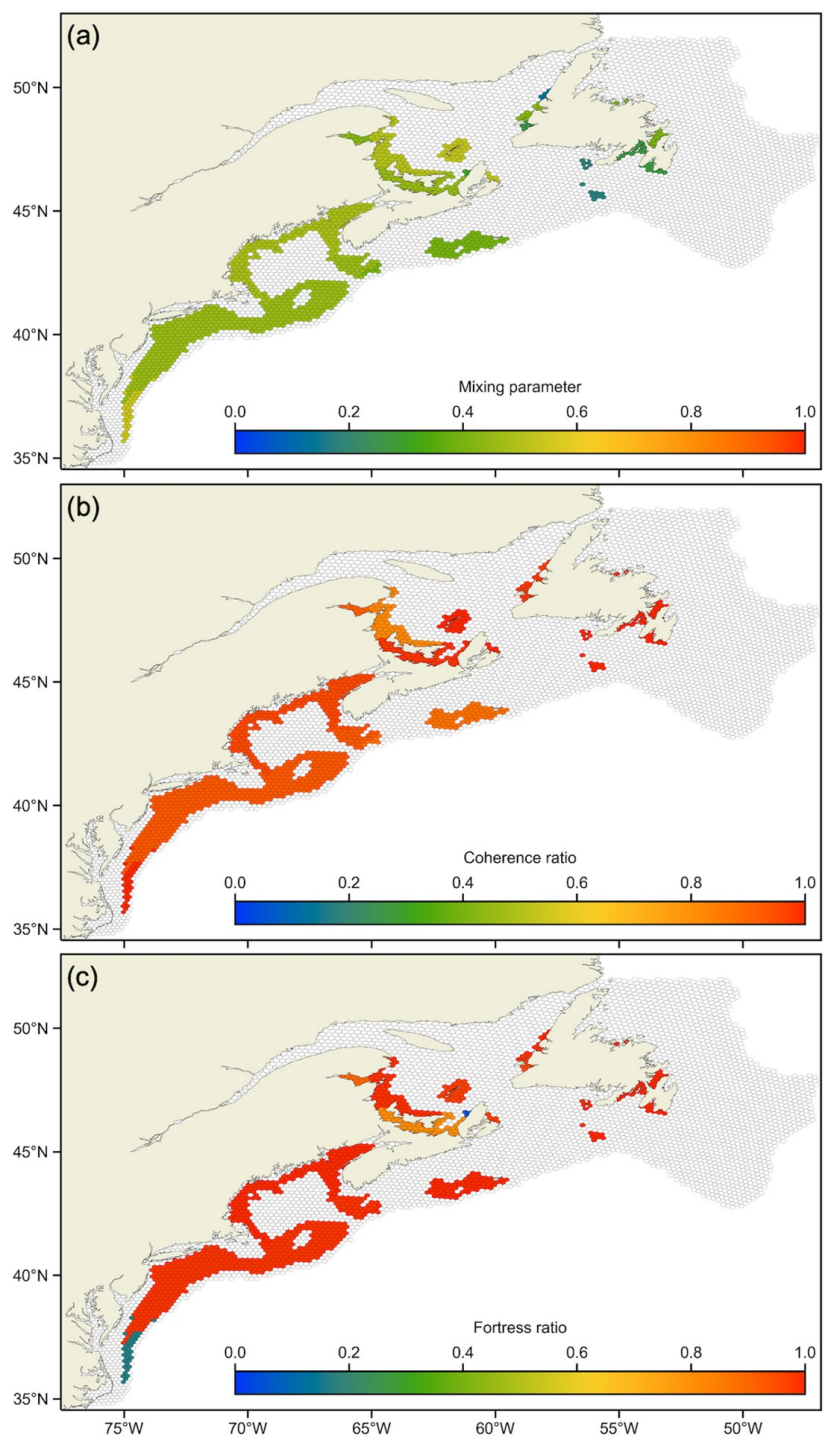
Spatial distribution of SH community quality. Community quality metrics (a) mixing parameter, (b) coherence ratio, and (c) fortress ratio. Bins are colored by the magnitude of the quality metric of the community to which they belong; those plotted with transparent faces were included in the model domain but do not belong to any community as they had no particles that spawned within them survive.

Quality metrics for communities in the SH transport network are shown in Fig 4, with values presented in Table 1. We chose simply to classify a community as a source/sink if the difference between its coherence and fortress ratio was at least 0.05. If both its coherence and fortress ratio were greater (lesser) than 0.95, the community was labelled impermeable (permeable).

Community quality metrics are relatively homogenous throughout the suitable case spatial structure. Averaging community quality metrics weighted by the number of bins in each community reveals the quality of the global network partition, resulting in a global mixing parameter equal to 0.45, a global coherence ratio equal to 0.93, and a global fortress ratio equal to 0.94.

**Table 1:** Quality metrics for each SH community. Proposed classifications were per Fig 2, using a 5% significance level. Communities are numbered from south to north, within each genetic lineage first.

Community	Community quality			Classification
	Mixing	Coherence	Fortress	
1	0.54	1.00	0.17	Sink
2	0.46	0.93	0.98	Source
3	0.48	0.95	1.00	Source
4	0.39	0.88	1.00	Source
5	0.18	1.00	1.00	Impermeable
6	0.45	0.98	0.81	Sink
7	0.52	1.00	0.97	Impermeable
8	0.50	0.82	0.99	Source
9	0.29	1.00	0.06	Sink
10	0.42	0.91	0.90	Permeable
11	0.26	1.00	1.00	Impermeable
12	0.40	1.00	1.00	Impermeable
13	0.25	0.97	0.95	Impermeable
14	0.42	0.98	0.98	Impermeable
15	0.14	0.97	1.00	Impermeable

**Fig 5:**
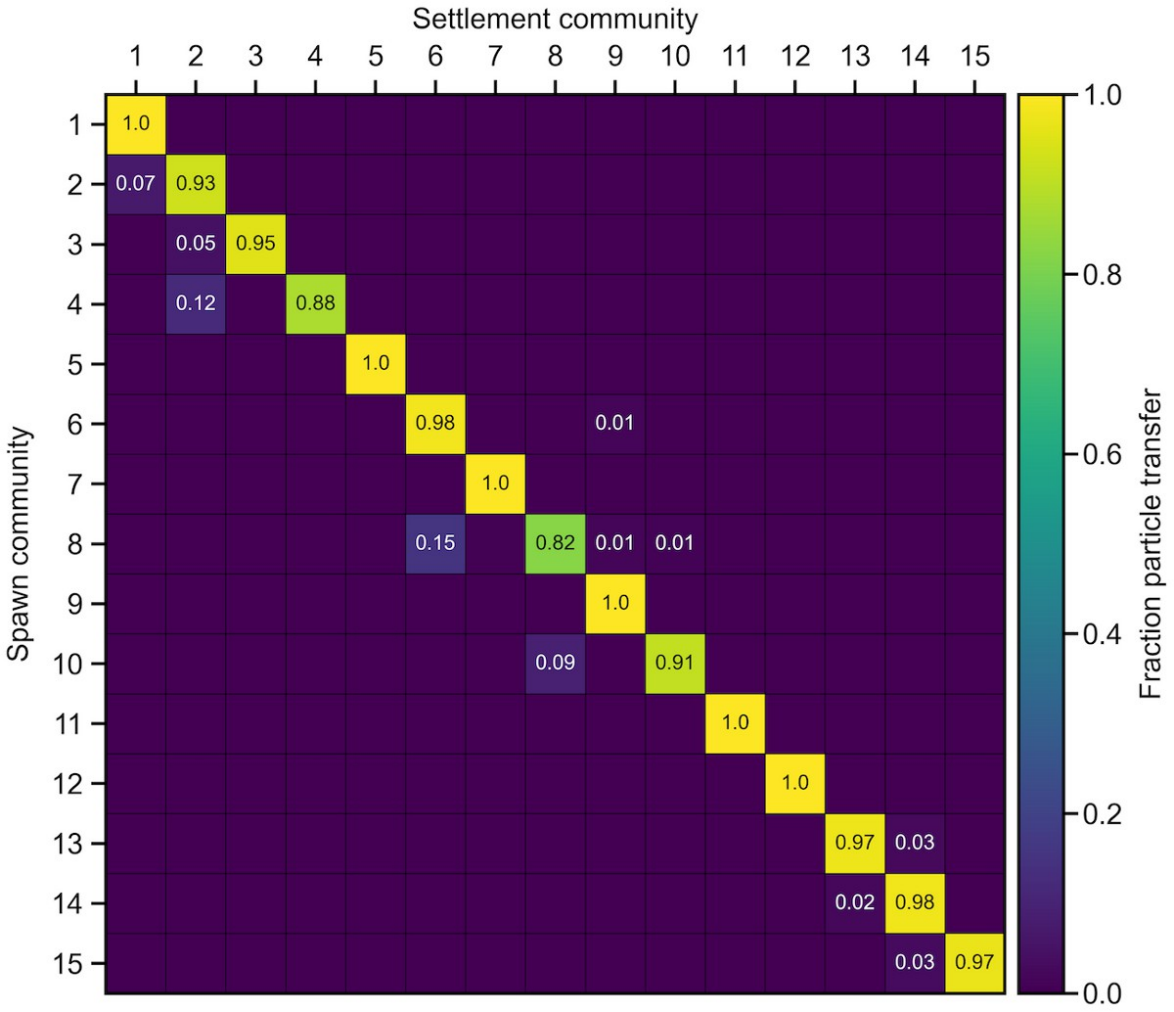
Connectivity matrix. Proportions (rounded) of surviving spawned particles that transitioned among communities in the SH case. Instances where no particles transitioned from one community to another are not labelled. Genetic lineages are indicated by bold white grid lines.

The SH connectivity matrix (Fig 5) confirms that there are few communities with significant (at least 5%) monodirectional particle exchange: communities 2 to 1, 3 to 2, and 4 to 2 within the southern lineage, and communities 8 to 6 and 10 to 8 within the Gulf of St. Lawrence lineage. Communities in the Newfoundland lineage (11–15) exchange few particles among themselves.

## Discussion

Our study investigated representing larval dispersal as a network and using community detection to demarcate subpopulations with high internal connectivity and sparse connectivity with other subpopulations. Our results show that including biological factors in particle tracking simulations, such as vertical migratory behaviour and the distribution of suitable habitat, affects community structure of the resultant transport network, i.e., *P. magellanicus* subpopulations. Spatial structure detected by *Infomap* in our three simulation scenarios contain similarities, but they ultimately differ topologically (Fig 3). The SH scenario, which incorporated the most extensive biological description of *P. magellanicus* based on extensive research including stock surveys, genetic population structure, and modelling, revealed metapopulation spatial substructure at a finer scale than was previously characterized.

### Spatial structure with modelled biology

While previous studies have applied *Infomap* to transport networks of marine populations, there has been limited consideration of how biological parameterization influences community structure. Our three simulation scenarios contain incremental differences in modelled biology that are known to affect connectivity of *P. magellanicus*: vertical distribution [26, 28] and suitable habitat [7, 26, 27]. The FD and PD cases have spatial structure that is different in regions where there is vertical shear in the flow such that the advection of larvae in the near-surface and the mixed layer is appreciably different. Notably, while communities occupying estuaries are similar in both cases, FD communities extend further out from the head due to greater estuarine-coastal exchange. The SH case differs substantially from the other two due to the profound influence of suitable habitat on spawn and successful settlement distributions. Even so, SH community structure partitions PD community structure; that is, each SH community (with only one single exception) approximately fits spatially within one and only one PD community. This does not happen by necessity because the SH transport network is constructed before community detection is performed. Thus, the similarity of community structure is due to consistent features in the flow that drive particle dispersal being represented in both transport networks.

### Comparison to genetics

Our implementation of suitable habitat dictates that each *P. magellanicus* subpopulation (community) is necessarily contained within one and only one genetic lineage, i.e., there is no gene flow among distinct genetic lineages in our model. The spatial structure presented here is at a finer scale than these genetic lineages. As such, this modelling approach reveals metapopulation substructure that is masked by exchange leading to genetic similarity.

For the most part, we see subpopulations within each genetic lineage exchanging larvae. Surprisingly though, we find some SH communities—communities 5, 11, and 12—that do not exchange with any other communities (i.e., they have unity coherence and fortress ratios). These three communities are around Newfoundland, with community 5 grouping with the southern lineage and communities 11 and 12 grouping with the Newfoundland lineage. Samples at sites within these communities exhibited significant genetic divergence from each other [5], suggesting there must only be a small extent of larval exchange with these subpopulations per generation that is not captured in our model.

There are several reasons to expect differences between patterns of community structure that emerge from our modelling approach and those derived from genetics. For example, our particle tracking simulation did not account for how variation in spawn time, spawn location, or temperature could affect PLD. Nor did it consider how spatially-varying larval mortality and larval production could alter transport networks by changing relative transfer [7]. Results here are only for an autumn spawn in September, whereas the seasonality of the dominant spawn varies geographically [35] and in some regions occurs semi-annually [53–55]. Moreover, the hydrodynamic fields used to drive physical transport processes were climatological, suggesting that interannual variations or extreme events could alter transport pathways and are not represented; higher frequency of model outputs and tides could further improve this study. With no coastal suitable habitat defined by the climatological species distribution model, our detected metapopulation spatial substructure represents climatological off-shore *P. magellanicus* subpopulations. Furthermore, future work would benefit from quantification of how detected community structure is robust to perturbations [46, 56].

### Analyzing connectivity

The suitable case global quality metric values reported in this study are comparable to previous studies in the case of the mixing parameter and notably higher in the case of the coherence ratio [16, 17], which suggests strong community structure in the SH transport network. Our fortress ratio informs about the proportion of settled larvae that had spawned within that same community, which together with the coherence ratio provides a holistic view of connectivity. For example, the southern-most SH community (1) has a unity coherence ratio but an irregularly low fortress ratio of 0.17, classifying it as a sink community. The connectivity matrix indicates this community receives 7% of the spawned particles from community 2. Within community 2, larvae are transported from Browns Bank into Georges Bank, and from Georges Bank into the southern reaches of the community in the Mid-Atlantic Bight [26, 28]; the along-shelf current that drives this dispersal varies considerably inter-annually [26, 28], similar to episodic connections from Browns Bank to Georges Bank [57]. These pathways are captured by our climatological hydrodynamic fields, resulting in the relatively large community 2. It becomes clear, then, that community 1 acts as a sink for the southbound particles transporting beyond the community 2 delineation. The boundary between communities 1 and 2 corresponds to a change in hydrodynamic flow from along shelf south to offshore [32, 58] as captured by its unity coherence ratio and high mixing parameter (0.54).

As is traditionally done to analyze connectivity, examination of the connectivity matrix constructed for the detected communities (Fig 5) reveals that these communities have high self-retention (i.e., large values along the main diagonal). The same conclusion can be drawn from the community coherence ratios (Table 1). The connectivity matrix also reveals some exchange among communities (i.e., off-diagonal elements), for example in the Gulf of St. Lawrence metapopulation (suitable case communities 6–10) with community 8 sourcing particles to community 6, and community 10 sourcing particles to community 8. The Gulf of St. Lawrence metapopulation consists of sink communities 6 and 9 (coherence ratio high; fortress ratio low), a source community 8 (low; high), an impermeable community 7 (high; high), and relatively permeable community 10 (low; low). Community 9 stands out as having an anomalously poor fortress ratio (0.06). Its detection as a distinct community may be explained by its relative size: only a small portion of particles transitioning from other communities (1% of spawned particles in community 6 and 8 each) to community 9 dramatically reduces its fortress ratio because few retained particles spawned within it. This, in addition to its unity coherence ratio, results in community 9 being detected as a distinct community instead of grouping with others. As exemplified here, we may characterize community-level dynamics and understand detected community structure using quality metrics and a connectivity matrix.

Previous modelling studies on Georges Bank considered *P. magellanicus* subpopulations on the Southern Flank, Northeast Peak, and Great South Channel based on tow data and international borders [7, 26, 27]. At the spatial scale we analyze (corresponding to a Markov time equal to 2 used by *Infomap*), these subpopulations are found to mutually group within SH community 2. We find that when using a smaller spatial scale (Markov time equal to 1), however, communities on Georges Bank are more comparable to those used in previous studies, albeit discontiguous, with fragments of the communities appearing in the Mid-Atlantic Bight, Gulf of Maine, and Browns Bank S1 Appendix. We leave to future studies the investigation of smaller-scale spatial structure in this region.

## Conclusions

We showed that the *Infomap* algorithm is well-suited for producing synoptic views of marine population spatial structure emerging from particle-track models considering vertical motility, individually-varying dispersal times, and heterogenous suitable habitat. This approach addresses limitations of previous connectivity studies for which spatial structure assessment was constrained to testing exchange among predefined subpopulations. Here, using network theory to summarize particle dispersal as a transport network, we computationally analyzed particle dispersal to reveal emergent spatial structure. Each community is classified as a sink, source, permeable, or impermeable using our introduced fortress ratio along with the existing coherence ratio.

Results for our model species *P. magellanicus* were generally consistent with previous studies, for instance highlighting high connectivity on Georges Bank and Mid-Atlantic Bight. We also identified spatial structure at a finer scale than indicated by genetic lineages, suggesting that gene flow is masking functional connectivity introduced by advection. The Gulf of St. Lawrence genetic lineage, for example, is found to consist of five subpopulations that are classified as being a sink, source, permeable, or impermeable using quality metrics. This has implications for predicting changes in *P. magellanicus* metapopulation dynamics.

With such a framework capable of characterizing spatial structure, we recommend its use for easily recognizing and describing changes in marine connectivity given different biophysical conditions. This approach can be used to assess the role of environmental variability and larval behaviours on spatial structure. Of particular interest is its application to understand the effects of climate change on marine population spatial structure by way of ocean circulation changes, the rising of average ocean temperature, and changes to suitable habitat. We have demonstrated the efficacy of this approach for the purpose of informing management efforts.

## Supporting information

S1 Fig Spatial distribution of FD community quality. Community quality metrics (a) mixing parameter, (b) coherence ratio, and (c) fortress ratio. Bins are colored by the magnitude of the quality metric of the community to which they belong; those plotted with transparent faces were included in the model domain but do not belong to any community as they had no particles that spawned within them survive.
(TIFF)

S2 Fig Spatial distribution of PD community quality. Community quality metrics (a) mixing parameter, (b) coherence ratio, and (c) fortress ratio. Bins are colored by the magnitude of the quality metric of the community to which they belong; those plotted with transparent faces were included in the model domain but do not belong to any community as they had no particles that spawned within them survive.
(TIFF)

S3 FigSH spatial structure with varying Markov time.(a) t=1, (b) t=2, and (c) t=3. Bins are colored by the community to which they belong; those plotted with transparent faces were included in the model domain but do not belong to any community as they had no particles that spawned within them survive. Communities are indexed from south to north, within each genetic lineage first.(TIFF)

S4 Fig Boundary persistence of community structure over 100 random seeds. Cases (a) FD, (b) PD, and (c) SH. Bins are colored by the fraction of the 100 partitions in the solution ensemble in which they were labelled as a community boundary; those plotted with transparent faces for a particular network were included in the model domain but do not belong to any community as they had no particles that spawned within them survive.
(TIFF)

S1 AppendixVarying Markov time.Markov time t is a parameter tuning the scale of detected community structure by *Infomap* [47–49]. Throughout our analysis, we specified a Markov time t=2. This parameter may be chosen freely to yield spatial structure at a scale convenient for analyses [17]. This was chosen *ad hoc* by examining detected community structure for different Markov times for an arbitrary random seed that best optimizes the map equation to determine our spatial scale of interest (S3 Fig). We found that t=2 corresponded to a spatial scale yielding detailed substructure within genetic lineages, whereas t=3 was a more coarse picture, and t=1 was difficult to interpret with its large number of small, discontiguous communities. Note that t need not be an integer; generally, t>0.(PDF)

S2 Appendix Boundary sensitivity to community detection. It is important during analyses to ensure that the network partitions being examined are representative of the ensemble of solutions generated by *Infomap* over random seeds [51]. [17] propose assessing the persistence at which *Infomap* draws boundaries between communities. A bin is said to be a boundary bin if it is directly adjacent to at least one bin belonging to a different community than its own. The boundary persistence of a bin is then the fraction of solutions in the ensemble in which it is labelled as a boundary (Reijnders et al., 2021). We find that boundaries are quite persistent for all model cases (S4 Fig). This is particularly true for the SH case, but even boundaries in the FD and PD cases are persistent within a tight range of bins—much more precise than used for interpretation of network partitions. We see that boundaries are often shifted by just one bin, which can dramatically reduce boundary persistence; however, when taken in context of a tight band of semi-persistent boundary bins, this indicates the general topology of the network partition is persistent in the solution ensemble. These boundary persistence plots give us confidence that an arbitrarily-selected partition from among those that best optimize the map equation is representative of the solution ensemble for the purposes of our analyses. We note that, in the SH case, we have instances of communities that contain no boundary bins, and are also discontiguous. Clearly, boundary persistence cannot indicate the existence of degenerate solutions in the form of spatially-isolated fragments of the community grouping separately from each other; given that communities are well-mixed, this is generally not of concern. Future work may consider more sophisticated measures of partition similarity.
(PDF)

## References

[1] Cowen RK, Sponaugle S. Larval Dispersal and Marine Population Connectivity. Annual Review of Marine Science. 2009;1:443–466. doi: 10.1146/annurev.marine.010908.163757 21141044

[2] Van Wyngaarden M, Snelgrove P, DiBacco C, Hamilton L, Rodriguez-Ezpeleta N, Jeffery N, et al. Identifying patterns of dispersal, connectivity, and selection in the sea scallop, Placopecten magellanicus, using RAD-seq derived SNPs. Evolutionary Applications. 2016;10. doi: 10.1111/eva.12432 28035239 PMC5192885

[3] Jeffery NW, DiBacco C, Van Wyngaarden M, Hamilton LC, Stanley RRE, Bernier R, et al. RAD sequencing reveals genomewide divergence between independent invasions of the European green crab (Carcinus maenas) in the Northwest Atlantic. Ecology and Evolution. 2017;7:2513–2524. doi: 10.1002/ece3.2872 28428843 PMC5395438

[4] Van Wyngaarden M, Snelgrove PVR, DiBacco C, Hamilton LC, Rodríguez-Ezpeleta N, Zhan L, et al. Oceanographic variation influences spatial genomic structure in the sea scallop, Placopecten magellanicus. Ecology and Evolution. 2017;8:2824–2841. doi: 10.1002/ece3.3846 PMC5838053 29531698

[5] Lehnert SJ, DiBacco C, Van Wyngaarden M, Jeffery NW, Lowen JB, Sylvester EVA, et al. Fine-scale temperature-associated genetic structure between inshore and offshore populations of sea scallop (Placopecten magellanicus). Heredity. 2019;122:69–80. doi: 10.1038/s41437-018-0087-9 29773897 PMC6288113

[6] Krumhansl KA, Gentleman WC, Wu Y, Wang X, DiBacco C. Using larval connectivity to inform conservation management of the endemic and threatened Atlantic mud-piddock (Barnea truncata) in the Minas Basin Canada. Frontiers in Marine Science. 2022;9. doi: 10.3389/fmars.2022.926442

[7] Davies KTA, Gentleman WC, DiBacco C, Johnson CL. Semi-annual spawning in marine scallops strengthens larval recruitment and connectivity on Georges Bank: A model study. Marine Ecology Progress Series. 2014;516:209–227. doi: 10.3354/meps10975

[8] Andrello M, Mouillot D, Beuvier J, Albouy C, Thuiller W, Manel S. Low Connectivity between Mediterranean Marine Protected Areas: A Biophysical Modeling Approach for the Dusky Grouper Epinephelus marginatus. PLOS ONE. 2013;8(7):1–15. doi: 10.1371/journal.pone.0068564 PMC3704643 23861917

[9] Haller G, Yuan G. Lagrangian coherent structures and mixing in two-dimensional turbulence. Physica D: Nonlinear Phenomena. 2000;147:352–370. doi: 10.1016/S0167-2789(00)00142-1

[10] Wichmann D, Delandmeter P, van Sebille E. Influence of Near-Surface Currents on the Global Dispersal of Marine Microplastic. Journal of Geophysical Research: Oceans. 2019;124:6086–6096. doi: 10.1029/2019JC015328 PMC6559306 31218155

[11] Rossi V, Ser-Giacomi E, Lõpez C, Hernández-García E. Hydrodynamic provinces and oceanic connectivity from a transport network help designing marine reserves. Geophysical Research Letters. 2014;41:2883–2891. doi: 10.1002/2014GL059540

[12] Rosvall M, Bergstrom CT. Maps of random walks on complex networks reveal community structure. Proceedings of the National Academy of Sciences. 2008;105:1118–1123. doi: 10.1073/pnas.0706851105 18216267 PMC2234100

[13] Girvan M, Newman MEJ. Community structure in social and biological networks. Proceedings of the National Academy of Sciences. 2002;99(12):7821–7826. doi: 10.1073/pnas.122653799 12060727 PMC122977

[14] Fortunato S. Community detection in graphs. Physics Reports. 2010;486(3):75–174. doi: 10.1016/j.physrep.2009.11.002

[15] Farage C, Edler D, Eklöf A, Rosvall M, Pilosof S. Identifying flow modules in ecological networks using Infomap. Methods in Ecology and Evolution. 2021;12(5):778–786. doi: 10.1111/2041-210X.13569

[16] Ser-Giacomi E, Rossi V, López C, Hernández-García E. Flow networks: A characterization of geophysical fluid transport. Chaos: An Interdisciplinary Journal of Nonlinear Science. 2015;25(3):036404. doi: 10.1063/1.4908231 25833442

[17] Reijnders D, van Leeuwen EJ, van Sebille E. Ocean Surface Connectivity in the Arctic: Capabilities and Caveats of Community Detection in Lagrangian Flow Networks. Journal of Geophysical Research: Oceans. 2021;126.

[18] Deauna JDL, Yatco KMB, Villanoy CL, Juinio-Meñez MA. Identification of Priority Sites to Support Management of Commercially Important Sea Cucumber Species by Applying Infomap and Habitat Filters to Larval Dispersal Data. Frontiers in Marine Science. 2021;7. doi: 10.3389/fmars.2020.571712

[19] Bharti DK, Guizien K, Aswathi-Das MT, Vinayachandran PN, Shanker K. Connectivity networks and delineation of disconnected coastal provinces along the Indian coastline using large-scale Lagrangian transport simulations. Limnology and Oceanography. 2022;67:1416–1428. doi: 10.1002/lno.12092

[20] Dubois M, Rossi V, Ser-Giacomi E, Arnaud-Haond S, López C, Hernández-García E. Linking basin-scale connectivity, oceanography and population dynamics for the conservation and management of marine ecosystems. Global Ecology and Biogeography. 2016;25(5):503–515. doi: 10.1111/geb.12431

[21] Bray L, Kassis D, Hall-Spencer JM. Assessing larval connectivity for marine spatial planning in the Adriatic. Marine Environmental Research. 2017;125:73–81. doi: 10.1016/j.marenvres.2017.01.006 28187325

[22] Pastor-Rollan A, Larsen J, Maar M, Hansen F. Agent-based modeling and genetics reveal the Limfjorden, Denmark, as a well-connected system for mussel larvae. Marine Ecology Progress Series. 2021. doi: 10.3354/meps13559

[23] Posgay JA. The range of sea scallop. Nautilus. 1957;71:55–57.

[24] Naidu KS, Robert G. In: Shumway SE, Parsons GJ, editors. Chapter 15 Fisheries sea scallop, Placopecten magellanicus. vol. 35. Elsevier; 2006. p. 869–905.

[25] Lowen JB, Hart DR, Stanley RRE, Lehnert SJ, Bradbury IR, DiBacco C, et al. Assessing effects of genetic, environmental, and biotic gradients in species distribution modelling. ICES Journal of Marine Science. 2019;76:1762–1775. doi: 10.1093/icesjms/fsz049

[26] Tian RC, Chen C, Stokesbury KDE, Rothschild BJ, Cowles GW, Xu Q, et al. Dispersal and settlement of sea scallop larvae spawned in the fishery closed areas on Georges Bank. ICES Journal of Marine Science. 2009;66(10):2155–2164. doi: 10.1093/icesjms/fsp175

[27] Gilbert CS, Gentleman WC, Johnson CL, DiBacco C, Pringle JM, Chen C. Modelling dispersal of sea scallop (Placopecten magellanicus) larvae on Georges Bank: The influence of depth-distribution, planktonic duration and spawning seasonality. Progress in Oceanography. 2010;87:37–48. doi: 10.1016/j.pocean.2010.09.021

[28] Chen C, Zhao L, Gallager S, Ji R, He P, Davis C, et al. Impact of Larval Behaviors on Dispersal and Connectivity of Sea Scallop Larvae over the Northeast U.S. Shelf. Progress in Oceanography. 2021;195:102604.

[29] Wang Z, Lu Y, Greenan B, Brickman D, DeTracey B. BNAM: An eddy-resolving North Atlantic Ocean model to support ocean monitoring. Can Tech Rep Hydrogr Ocean Sci. 2018;327: vii + 18p.

[30] Delandmeter P, van Sebille E. The Parcels v2.0 Lagrangian framework: new field interpolation schemes. Geoscientific Model Development. 2019;12:3571–3584. doi: 10.5194/gmd-12-3571-2019

[31] Tremblay MJ, Loder JW, Werner FE, Naimie CE, Page FH, Sinclair MM. Drift of sea scallop larvae Placopecten magellanicus on Georges Bank: a model study of the roles of mean advection, larval behavior and larval origin. Deep Sea Research Part II: Topical Studies in Oceanography. 1994;41(1):7–49. doi: 10.1016/0967-0645(94)90061-2

[32] Krumhansl K, Gentleman W, Lee K, Ramey-Balci P, Goodwin J, Wang Z, et al. Permeability of coastal biogeographic barriers to marine larval dispersal on the east and west coasts of North America. Global Ecology and Biogeography. 2023;32(6):945–961. doi: 10.1111/geb.13654

[33] Chia FS, Buckland-Nicks J, Young C. Locomotion of marine invertebrate larvae: A review. Canadian Journal of Zoology. 1984;62:1205–1222. doi: 10.1139/z84-176

[34] Tremblay MJ, Sinclair M. Planktonic Sea Scallop Larvae (Placopecten magellanicus) in the Georges Bank Region: Broadscale Distribution in Relation to Physical Oceanography. Canadian Journal of Fisheries and Aquatic Sciences. 1992;49:1597–1615. doi: 10.1139/f92-178

[35] Barber BJ, Blake NJ. In: Shumway SE, Parsons GJ, editors. Chapter 6 Reproductive Physiology. vol. 35. Elsevier; 2006. p. 357–416.

[36] Uber. H3: Uber’s Hexagonal Hierarchical Spatial Index; 2018. Available from: https://eng.uber.com/h3/.

[37] O’Malley M, Sykulski AM, Laso-Jadart R, Madoui MA. Estimating the Travel Time and the Most Likely Path from Lagrangian Drifters. Journal of Atmospheric and Oceanic Technology. 2021;38:1059–1073. doi: 10.1175/JTECH-D-20-0134.1

[38] Molkenthin N, Rehfeld K, Marwan N, Kurths J. Networks from Flows—From Dynamics to Topology. Scientific Reports. 2014;4. doi: 10.1038/srep04119 24535026 PMC3927207

[39] Strogatz SH. Exploring complex networks. Nature. 2001;410:268–276. doi: 10.1038/35065725 11258382

[40] Newman M, Barabási AL, Watts DJ. The Structure and Dynamics of Networks:. Princeton Studies in Complexity. Princeton University Press; 2006.

[41] Froyland G, Stuart RM, van Sebille E. How well-connected is the surface of the global ocean? Chaos: An Interdisciplinary Journal of Nonlinear Science. 2014;24(3):033126. doi: 10.1063/1.4892530 25273206

[42] Bohlin L, Edler D, Lancichinetti A, Rosvall M. In: Ding Y, Rousseau R, Wolfram D, editors. Community Detection and Visualization of Networks with the Map Equation Framework. Springer International Publishing; 2014. p. 3–34.

[43] Edler D, Bohlin L, Rosvall M. Mapping Higher-Order Network Flows in Memory and Multilayer Networks with Infomap. Algorithms. 2017;10(4). doi: 10.3390/a10040112

[44] Fortunato S, Barthélemy M. Resolution limit in community detection. Proceedings of the National Academy of Sciences. 2007;104:36–41. doi: 10.1073/pnas.0605965104 17190818 PMC1765466

[45] Kawamoto T, Rosvall M. Estimating the resolution limit of the map equation in community detection. Phys Rev E. 2015;91:012809. doi: 10.1103/PhysRevE.91.012809 25679659

[46] Rosvall M, Bergstrom CT. Mapping Change in Large Networks. PLOS ONE. 2010;5(1):1–7. doi: 10.1371/journal.pone.0008694 20111700 PMC2811724

[47] Kheirkhahzadeh M, Lancichinetti A, Rosvall M. Efficient community detection of network flows for varying Markov times and bipartite networks. Phys Rev E. 2016;93:032309. doi: 10.1103/PhysRevE.93.032309 27078368

[48] Schaub MT, Delvenne JC, Yaliraki SN, Barahona M. Markov Dynamics as a Zooming Lens for Multiscale Community Detection: Non Clique-Like Communities and the Field-of-View Limit. PLOS ONE. 2012;7(2):1–11. doi: 10.1371/journal.pone.0032210 22384178 PMC3288079

[49] Schaub MT, Lambiotte R, Barahona M. Encoding dynamics for multiscale community detection: Markov time sweeping for the map equation. Phys Rev E. 2012;86:026112. doi: 10.1103/PhysRevE.86.026112 23005830

[50] Lambiotte R, Rosvall M. Ranking and clustering of nodes in networks with smart teleportation. Phys Rev E. 2012;85:056107. doi: 10.1103/PhysRevE.85.056107 23004821

[51] Calatayud J, Bernardo-Madrid R, Neuman M, Rojas A, Rosvall M. Exploring the solution landscape enables more reliable network community detection. Physical Review E. 2019;100:52308. doi: 10.1103/PhysRevE.100.052308 31869919

[52] Peel L, Larremore DB, Clauset A. The ground truth about metadata and community detection in networks. Science Advances. 2017;3(5):e1602548. doi: 10.1126/sciadv.1602548 28508065 PMC5415338

[53] DuPaul WD, Kirkley JE, Schmitzer AC. Evidence of a semiannual reproductive cycle for the sea scallop, Placopecten magellanicus (Gemlin 1971), in the mid-Atlantic region. Journal of Shellfish Research. 1989;8:173–178.

[54] Schmitzer AC, DuPaul WD, Kirkley JE. Gametogenic cycle of sea scallops Placopecten magellanicus (Gmelin, 1791) in the mid-Atlantic Bight. Journal of Shellfish Research. 1991;10:221–228.

[55] DiBacco C, Robert G, Grant J. Reproductive cycle of the sea scallop, Placopecten magellanicus (Gemlin 1971) on northeastern Georges Bank. Journal of Shellfish Research. 1995;14:56–59.

[56] Karrer B, Levina E, Newman MEJ. Robustness of community structure in networks. Phys Rev E. 2008;77:046119. doi: 10.1103/PhysRevE.77.046119 18517702

[57] Smith PC, Flagg CN, Limeburner R, Fuentes-Yaco C, Hannah C, Beardsley RC, et al. Scotian shelf crossovers during winter/spring 1999. Journal of Geophysical Research: Oceans. 2003;108. doi: 10.1029/2001JC001288

[58] Roarty H, Glenn S, Brodie J, Nazzaro L, Smith M, Handel E, et al. Annual and Seasonal Surface Circulation Over the Mid-Atlantic Bight Continental Shelf Derived From a Decade of High Frequency Radar Observations. Journal of Geophysical Research: Oceans. 2020;125:e2020JC016368. doi: 10.1029/2020JC016368

