# Supplementary figures and images for: Assessing spatial structure in marine populations using network theory: A case study of Atlantic sea scallop (Placopecten magellanicus) connectivity
Source: PLoS One. 2024 Nov 13;19(11):e0308787. doi: 10.1371/journal.pone.0308787 (PMC11559974; doi:10.1371/journal.pone.0308787)

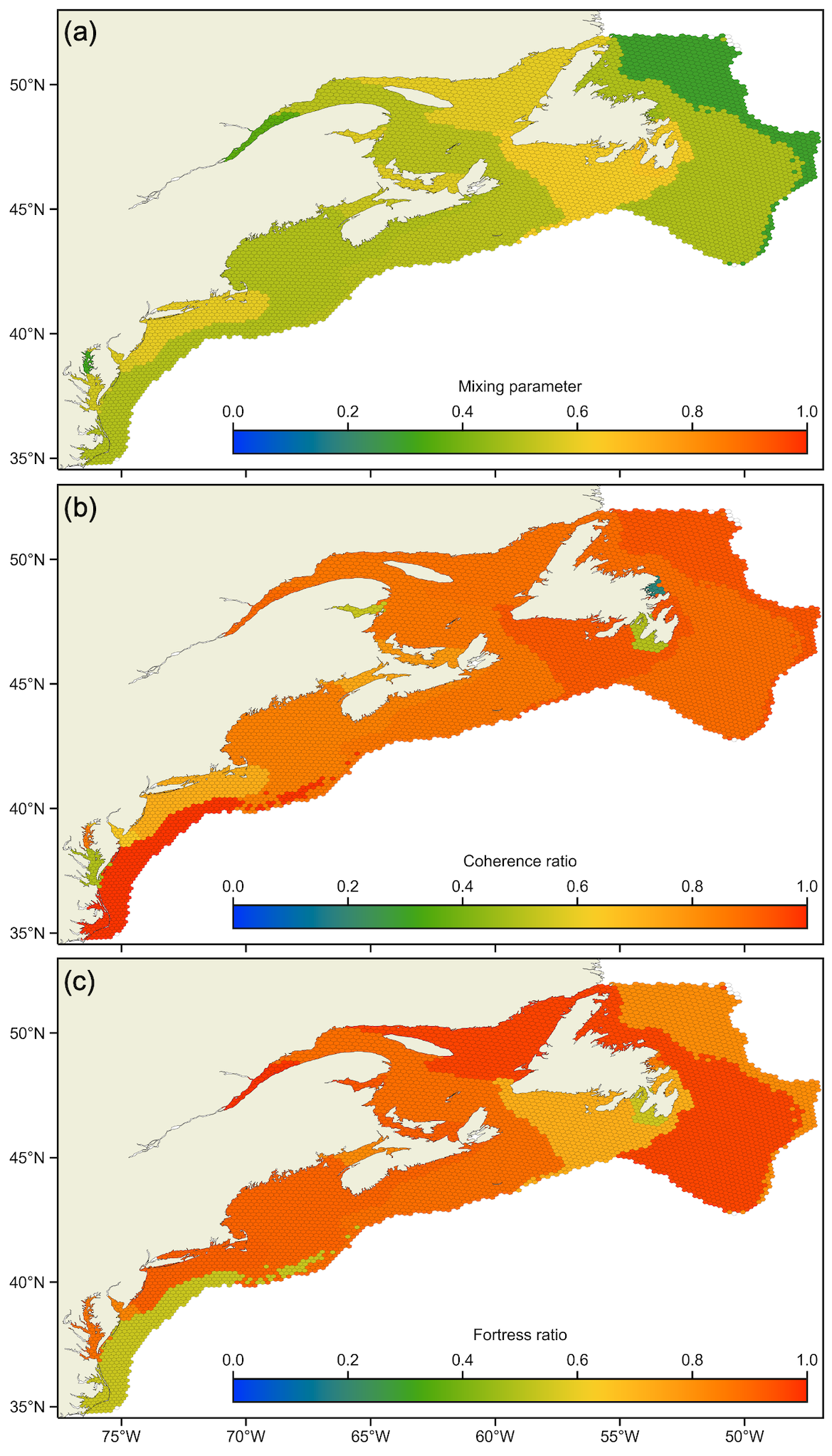

Supplement: S1 Fig — Community quality metrics (a) mixing parameter, (b) coherence ratio, and (c) fortress ratio. Bins are colored by the magnitude of the quality metric of the community to which they belong; those plotted with transparent faces were included in the model domain but do not belong to any community as they had no particles that spawned within them survive. (TIFF) [file pone.0308787.s001.tiff]

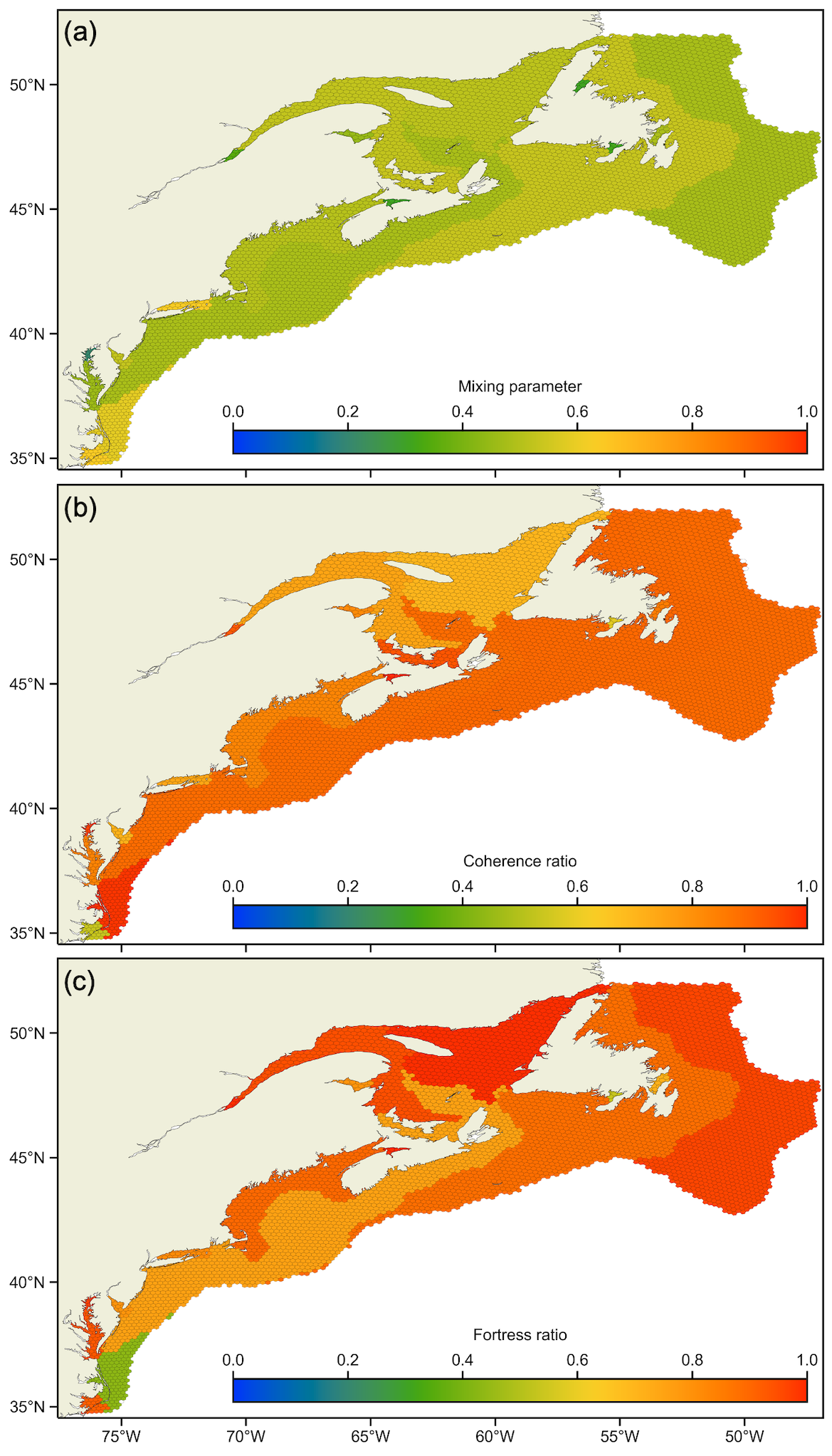

Supplement: S2 Fig — Community quality metrics (a) mixing parameter, (b) coherence ratio, and (c) fortress ratio. Bins are colored by the magnitude of the quality metric of the community to which they belong; those plotted with transparent faces were included in the model domain but do not belong to any community as they had no particles that spawned within them survive. (TIFF) [file pone.0308787.s002.tiff]

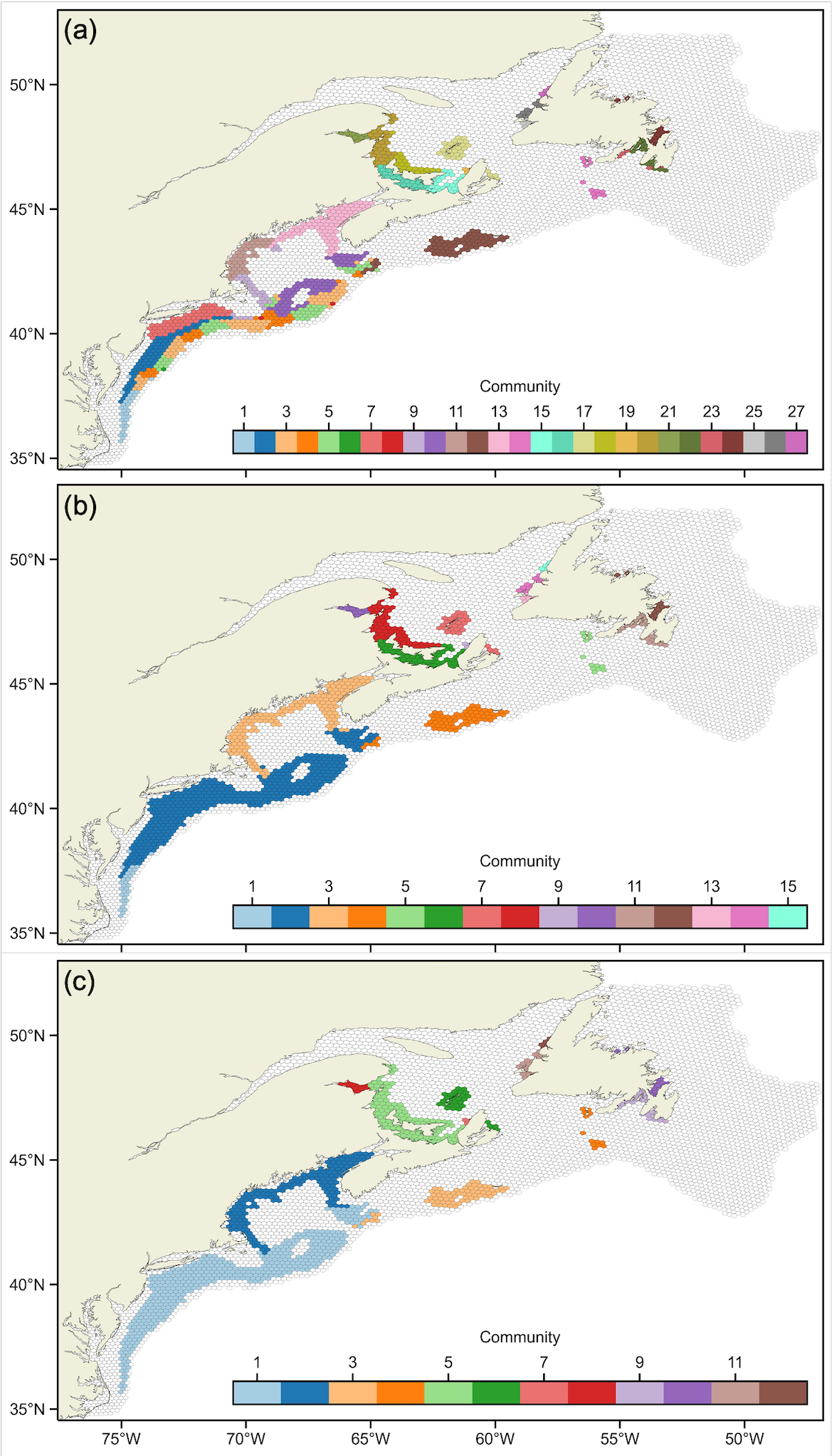

Supplement: S3 Fig — (a) t=1, (b) t=2, and (c) t=3. Bins are colored by the community to which they belong; those plotted with transparent faces were included in the model domain but do not belong to any community as they had no particles that spawned within them survive. Communities are indexed from south to north, within each genetic lineage first. (TIFF) [file pone.0308787.s003.tiff]

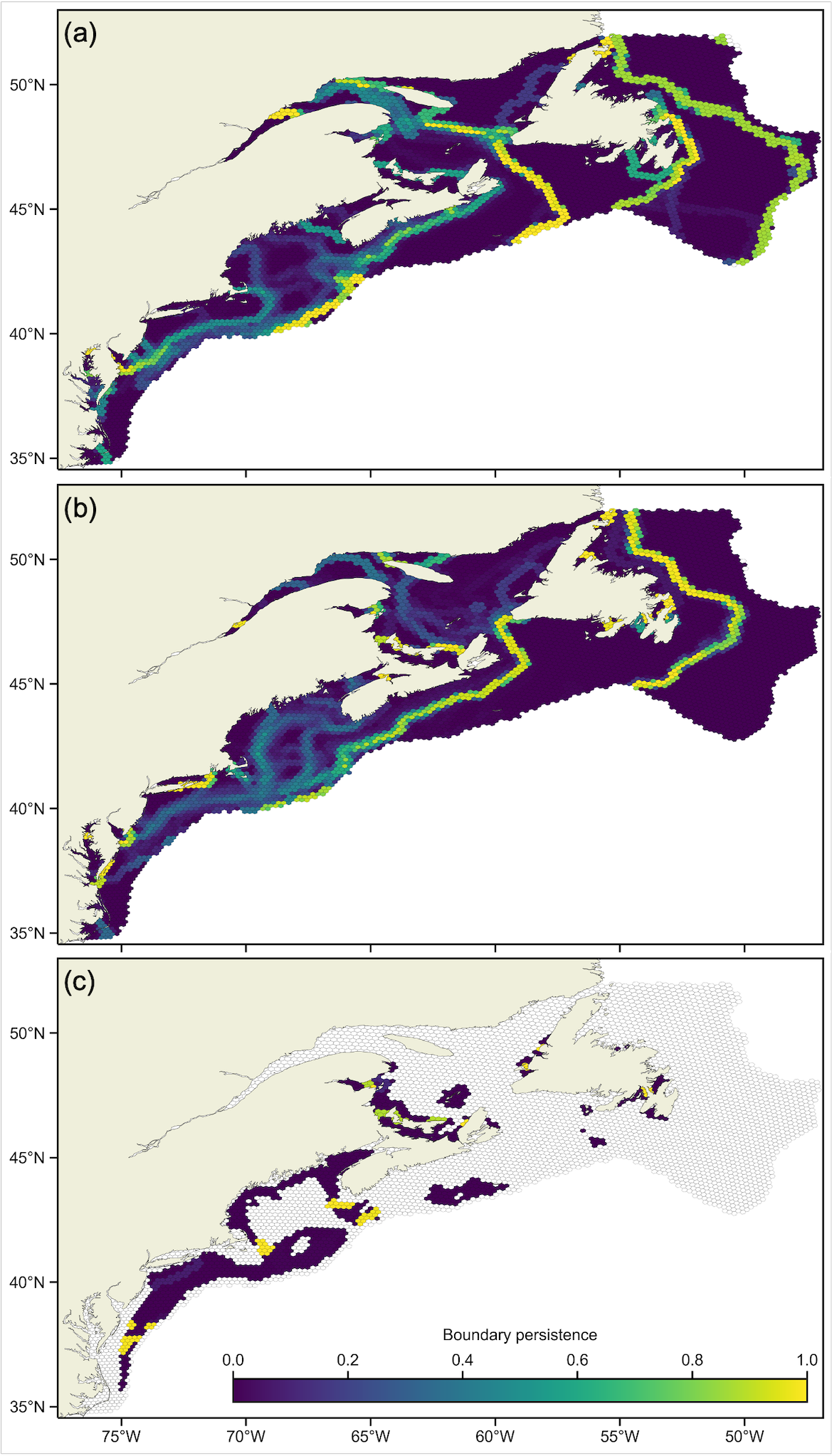

Supplement: S4 Fig — Cases (a) FD, (b) PD, and (c) SH. Bins are colored by the fraction of the 100 partitions in the solution ensemble in which they were labelled as a community boundary; those plotted with transparent faces for a particular network were included in the model domain but do not belong to any community as they had no particles that spawned within them survive. (TIFF) [file pone.0308787.s004.tiff]
