# Supplementary material for: Assessing spatial structure in marine populations using network theory: A case study of Atlantic sea scallop (Placopecten magellanicus) connectivity
Source: PLoS One. 2024 Nov 13;19(11):e0308787. doi: 10.1371/journal.pone.0308787 (PMC11559974; doi:10.1371/journal.pone.0308787)
Supplement: S1 Appendix — Markov time t is a parameter tuning the scale of detected community structure by Infomap [47–49]. Throughout our analysis, we specified a Markov time t=2. This parameter may be chosen freely to yield spatial structure at a scale convenient for analyses [17]. This was chosen ad hoc by examining detected community structure for different Markov times for an arbitrary random seed that best optimizes the map equation to determine our spatial scale of interest (S3 Fig). We found that t=2 corresponded to a spatial scale yielding detailed substructure within genetic lineages, whereas t=3 was a more coarse picture, and t=1 was difficult to interpret with its large number of small, discontiguous communities. Note that t need not be an integer; generally, t>0. (PDF) [file pone.0308787.s005.pdf]

S1 Appendix. Varying Markov time. Markov time $\texttt{t}$ is a parameter tuning the scale of detected community structure by *Infomap* [2–4]. Throughout our analysis, we specified a Markov time $\texttt{t} = 2$. This parameter may be chosen freely to yield spatial structure at a scale convenient for analyses [1]. This was chosen *ad hoc* by examining detected community structure for different Markov times for an arbitrary random seed that best optimizes the map equation to determine our spatial scale of interest (S3 Fig). We found that $\texttt{t} = 2$ corresponded to a spatial scale yielding detailed substructure within genetic lineages, whereas $\texttt{t} = 3$ was a more coarse picture, and $\texttt{t} = 1$ was difficult to interpret with its large number of small, discontiguous communities. Note that $\texttt{t}$ need not be an integer; generally, $\texttt{t} > 0$.

References

1. Reijnders D, van Leeuwen EJ, van Sebille E. Ocean Surface Connectivity in the Arctic: Capabilities and Caveats of Community Detection in Lagrangian Flow Networks. Journal of Geophysical Research: Oceans. 2021;126. doi:10.1029/2020JC016416.

2. Kheirkhahzadeh M, Lancichinetti A, Rosvall M. Efficient community detection of network flows for varying Markov times and bipartite networks. Phys Rev E. 2016;93:032309. doi:10.1103/PhysRevE.93.032309.

3. Schaub MT, Delvenne JC, Yaliraki SN, Barahona M. Markov Dynamics as a Zooming Lens for Multiscale Community Detection: Non Clique-Like Communities and the Field-of-View Limit. PLOS ONE. 2012;7(2):1–11. doi:10.1371/journal.pone.0032210.

4. Schaub MT, Lambiotte R, Barahona M. Encoding dynamics for multiscale community detection: Markov time sweeping for the map equation. Phys Rev E. 2012;86:026112. doi:10.1103/PhysRevE.86.026112.
