# Supplementary material for: Assessing spatial structure in marine populations using network theory: A case study of Atlantic sea scallop (Placopecten magellanicus) connectivity
Source: PLoS One. 2024 Nov 13;19(11):e0308787. doi: 10.1371/journal.pone.0308787 (PMC11559974; doi:10.1371/journal.pone.0308787)
Supplement: S2 Appendix — It is important during analyses to ensure that the network partitions being examined are representative of the ensemble of solutions generated by Infomap over random seeds [51]. [17] propose assessing the persistence at which Infomap draws boundaries between communities. A bin is said to be a boundary bin if it is directly adjacent to at least one bin belonging to a different community than its own. The boundary persistence of a bin is then the fraction of solutions in the ensemble in which it is labelled as a boundary (Reijnders et al., 2021). We find that boundaries are quite persistent for all model cases (S4 Fig). This is particularly true for the SH case, but even boundaries in the FD and PD cases are persistent within a tight range of bins—much more precise than used for interpretation of network partitions. We see that boundaries are often shifted by just one bin, which can dramatically reduce boundary persistence; however, when taken in context of a tight band of semi-persistent boundary bins, this indicates the general topology of the network partition is persistent in the solution ensemble. These boundary persistence plots give us confidence that an arbitrarily-selected partition from among those that best optimize the map equation is representative of the solution ensemble for the purposes of our analyses. We note that, in the SH case, we have instances of communities that contain no boundary bins, and are also discontiguous. Clearly, boundary persistence cannot indicate the existence of degenerate solutions in the form of spatially-isolated fragments of the community grouping separately from each other; given that communities are well-mixed, this is generally not of concern. Future work may consider more sophisticated measures of partition similarity. (PDF) [file pone.0308787.s006.pdf]

S2 Appendix. Boundary sensitivity to community detection. It is important during analyses to ensure that the network partitions being examined are representative of the ensemble of solutions generated by *Infomap* over random seeds [2]. [1] propose assessing the persistence at which *Infomap* draws boundaries between communities. A bin is said to be a boundary bin if it is directly adjacent to at least one bin belonging to a different community than its own. The boundary persistence of a bin is then the fraction of solutions in the ensemble in which it is labelled as a boundary [1]. We find that boundaries are quite persistent for all model cases (S4 Fig). This is particularly true for the SH case, but even boundaries in the FD and PD cases are persistent within a tight range of bins — much more precise than used for interpretation of network partitions. We see that boundaries are often shifted by just one bin, which can dramatically reduce boundary persistence; however, when taken in context of a tight band of semi-persistent boundary bins, this indicates the general topology of the network partition is persistent in the solution ensemble. These boundary persistence plots give us confidence that an arbitrarily-selected partition from among those that best optimize the map equation is representative of the solution ensemble for the purposes of our analyses. We note that, in the SH case, we have instances of communities that contain no boundary bins, and are also discontiguous. Clearly, boundary persistence cannot indicate the existence of degenerate solutions in the form of spatially-isolated fragments of the community grouping separately from each other; given that communities are well-mixed, this is generally not of concern. Future work may consider more sophisticated measures of partition similarity.

References

1. Reijnders D, van Leeuwen EJ, van Sebille E. Ocean Surface Connectivity in the Arctic: Capabilities and Caveats of Community Detection in Lagrangian Flow Networks. Journal of Geophysical Research: Oceans. 2021;126. doi:10.1029/2020JC016416.

2. Calatayud J, Bernardo-Madrid R, Neuman M, Rojas A, Rosvall M. Exploring the solution landscape enables more reliable network community detection. Physical Review E. 2019;100:52308. doi:10.1103/PhysRevE.100.052308.
